# The impact of an *ex vivo* paediatric renal replacement therapy circuit on antimicrobial concentrations

**DOI:** 10.1093/jac/dkag189

**Published:** 2026-06-03

**Authors:** Michele L Cree, Mohd Hafiz Abdul-Aziz, Steven C Wallis, Hayoung Won, Chandra D Sumi, Dusan Marjanovic, Jenny L Ordonez, Luregn J Schlapbach, Jason A Roberts

**Affiliations:** UQ Centre for Clinical Research, Faculty of Health, Medicine and Behavioural Sciences, The University of Queensland, Brisbane, Australia; Pharmacy Department, Queensland Children’s Hospital, Brisbane, Australia; UQ Centre for Clinical Research, Faculty of Health, Medicine and Behavioural Sciences, The University of Queensland, Brisbane, Australia; UQ Centre for Clinical Research, Faculty of Health, Medicine and Behavioural Sciences, The University of Queensland, Brisbane, Australia; UQ Centre for Clinical Research, Faculty of Health, Medicine and Behavioural Sciences, The University of Queensland, Brisbane, Australia; UQ Centre for Clinical Research, Faculty of Health, Medicine and Behavioural Sciences, The University of Queensland, Brisbane, Australia; UQ Centre for Clinical Research, Faculty of Health, Medicine and Behavioural Sciences, The University of Queensland, Brisbane, Australia; UQ Centre for Clinical Research, Faculty of Health, Medicine and Behavioural Sciences, The University of Queensland, Brisbane, Australia; UQ, Centre for Children’s Health Research, Brisbane, Australia; Department of Intensive Care and Neonatology, and Children`s Research Center, University Children's Hospital Zurich, University of Zurich, Zurich, Switzerland; UQ Centre for Clinical Research, Faculty of Health, Medicine and Behavioural Sciences, The University of Queensland, Brisbane, Australia; Department of Intensive Care Medicine, Royal Brisbane & Women’s Hospital, Brisbane, Australia; Herston Infectious Diseases Institute (HeIDI), Level 8, UQ Centre for Clinical Research Royal Brisbane and Women’s Hospital, Brisbane, Australia; Division of Anaesthesiology Critical Care Emergency and Pain Medicine, Nîmes University Hospital, University of Montpellier, Nîmes, France; Pharmacy Department, Royal Brisbane and Women’s Hospital, Brisbane, Australia

## Abstract

**Background:**

Critically ill children receiving continuous renal replacement therapy may experience sub-therapeutic concentrations for antimicrobials leading to treatment failure and antimicrobial resistant pathogens. The objective of this study was to determine whether antimicrobial concentrations are reduced by a paediatric continuous renal replacement therapy (CRRT).

**Method:**

An *ex vivo* closed continuous veno–venous haemodiafiltration was simulated for a 3 kg infant to assess antimicrobial clearance across three ultrafiltration rates (zero, low and high flux). Slow continuous ultrafiltration was used to assess antimicrobial adsorption and recovery over 240 minutes. Controls were included to account for spontaneous drug degradation. This study was conducted in a university research laboratory with no participants. Antimicrobial concentrations were measured using a validated HPLC-MS/MS method.

**Results:**

The antimicrobial filter clearance during high-flux filtration was significantly increased for fluconazole, piperacillin, tazobactam, vancomycin and voriconazole (*P* < 0.05). The antimicrobial recovery [mean (%)] at 240 minutes in the CRRT model was significantly different from baseline (time zero) for ampicillin 49%, fluconazole 76%, gentamicin (0%) meropenem 51%, piperacillin 54%, vancomycin 31% and voriconazole 47% (*P* < 0.05). A significant relationship was demonstrated between antimicrobial recovery and molecular charge (*R*^2^ = 0.58 *P* < 0.001) in the CRRT model; no relationships were reported for lipophilicity or protein binding.

**Conclusions:**

The concentrations were reduced in >70% of the study antimicrobials in the *ex vivo* paediatric CRRT model, as a result of an increase in filter clearance during high-flux filtration or from drug-circuit adsorption. These findings suggests that antimicrobial dosing in critically ill children receiving CRRT requires assessment to determine whether antimicrobial concentrations are therapeutic.

## Background

Children admitted to the intensive care unit (ICU) with severe infections and sepsis often either present with or develop an acute kidney injury (AKI) that may require extracorporeal renal support.^1,2^ Continuous renal replacement therapy (CRRT) is commonly used to provide extracorporeal renal support in this setting. Critically ill patients with a confirmed infection who receive CRRT have substantially lower long-term survival.^3,4^ CRRT comprises multiple components, including a blood pump, tubing, filter, as well as pre-blood pump, dialysis and replacement fluids, which collectively enable solute, electrolyte and fluid removal through diffusion and convection.^5^ Importantly, these mechanisms may also contribute to the removal of some antimicrobials (e.g. beta-lactams antibiotics), leading to sub-therapeutic concentrations.^6,7^

The extent of antimicrobial removal during CRRT is influenced by the drug’s physicochemical properties, including molecular weight and protein binding, as well as by the pore size of the dialysis membrane.^7^ Clinical studies in critically ill adult patients receiving CRRT have shown that antimicrobials with low molecular weight (<500 Daltons) are more likely to demonstrate an increased clearance,^8–10^ while highly protein bound antimicrobials tend to be less affected.^11^ Since many commonly used antimicrobials are renally eliminated, have low molecular weights and exhibit low to moderate protein binding (see Table 1^12–19^), the use of CRRT may increase the clearance and consequently reduce the concentrations of these antimicrobials.

**Table 1.** Drug physicochemical properties for the antimicrobials

Antimicrobial	Molecular weight (Daltons or g.mol^−1^)	Molecular charge	Protein binding (%)		log*P*	Route of elimination	Source
			Neonates	Child			
Ampicillin	349^13^	0^12,13^	10^14^	15 to 30^14^	1.4^16^	Primarily renal excretion^12^	Mylan Pty Ltd, Australia
Cefotaxime	455^13^	0^12,13^	NR	35 to 40^15^	−1.4^15,16^	70% renal elimination^15^	Hospira Pty Ltd, Australia
Flucloxacillin	454^13^	−1^12^	75^17^	95 to 97^17^	2.6^18^	60–76% renal elimination^19^	Juno Pharmaceuticals Pty Ltd, Australia
Fluconazole	306^13^	0^13^	NR	11 to 12^15^	0.5^15^	80% renal elimination^13,15^	Sigma Pty Ltd, Australia
Gentamicin	478^13^	5^12^	<10^15^	30^15,16^	−3.1^15^	90% renal elimination^15^	Pfizer Pty Ltd, Australia
Meropenem	383^13^	0^12^	NR	2^15,16^	− 0.7^15^	70% renal elimination^15^	Fresenius Kabi Pty Ltd, Germany
Micafungin	1270^13^	0^13^	90^15^	96^15^	−1.5^15^	83% excreted (12% urine, 71% faeces)^15^	Astellas Pharma Pty Ltd, Australia
Piperacillin	518^13^	−1 ^12^	30^15^	16 to 48^15^	0.5^15,16^	68% renal elimination^15^	AFT pharmaceuticals Pty Ltd, Australia
Tazobactam	300^13^	−1^12^	NR	30^15^	− 0.9^15,16^	80% renal elimination^12^	AFT pharmaceuticals Pty Ltd, Australia
Vancomycin	1449^13^	1^12^	10 to 20^15^	10 to 55^15,16^	−1.4^15^	80–90% renal elimination^15^	Sandoz Pty Ltd, Australia
Voriconazole	349^13^	0^13^	NR	60^15^	2.6^15^	< 2% renal, hepatic elimination through CYP2C19, CYP3A4, & CYP2C9^15^	Pfizer Pty Ltd, Australia

NR, not reported in the published literature.

Drug adsorption onto the CRRT circuit is an additional mechanism that may potentially reduce antimicrobial concentrations.^20^  *Ex vivo* studies using adult CRRT circuits have demonstrated adsorption-related losses for several antimicrobials, including ciprofloxacin,^21^ gentamicin,^22,23^ meropenem^21,24^ and vancomycin.^21,24,25^ Circuit-related factors, such as membrane surface area and composition (e.g. asymmetric triacetate haemofilter membrane), as well as the use of cytokine adsorbers, can influence the extent of adsorption and reduce antimicrobial concentrations.^21,24^

Several studies have reported sub-therapeutic antimicrobial concentrations in critically ill adults receiving CRRT,^4,26,27^ which may hinder effective elimination of pathogens and potentially lead to worse patient outcomes such as ICU length of stay and mortality.^4^ However, limited scientific evidence is available describing antimicrobial pharmacokinetics in critically ill children receiving CRRT.^15^ Antimicrobial dosing in this patient population remains challenging, often relying on clinical judgement due to the limited availability of therapeutic drug monitoring (TDM). These challenges may be magnified in critically ill children receiving antimicrobials and CRRT due to a potential for increased clearance or antimicrobial-circuit adsorption, resulting in reduced antimicrobial concentrations, treatment failure and/or the emergence of antimicrobial resistance.

To address this knowledge gap, an *ex vivo* study was performed using a paediatric CRRT model to evaluate whether commonly used antibiotics and antifungals are removed through increased clearance or adsorption.

## Method

### Institutional review board

This study did not involve human or animal subjects, institutional review board (IRB) approval was obtained for the blood products by the Children’s Health Queensland IRB and The University of Queensland IRB (STUDY66639, ‘Ex Vivo Characterisation of Paediatric Extracorporeal Therapy (Extracorporeal Membrane Oxygenations (ECMO) and Extracorporeal Continuous Renal Replacement Therapy (CRRT)) on Antimicrobials Concentrations’, approved 28 October 2020) was conducted in accordance with ethical standards of The University of Queensland IRB and the Helsinki Declaration of 1975.

### Ex vivo *continuous renal replacement therapy (CRRT) circuit setup*

#### Phase 1: extracorporeal clearance study

*Ex vivo* CRRT was performed using a Gambro Prismaflex machine (Baxter Ptd Ltd, Australia) and a blood–crystalloid mixture in continuous haemodiafiltration (CVVHDF) mode, incorporating a 0.6 m^2^ AN69 ST hollow-fibre haemofilter (Baxter Ptd Ltd, Australia) to simulate paediatric CRRT in a 3 kg infant. Blood products (<5 days old) were obtained through a material supply agreement with the Australian Red Cross Life-Blood Pty Limited. The blood–crystalloid mixture was prepared in a polyvinyl chloride (PVC) jar by mixing 300 mL of human whole blood (pseudo patient) with 695 mL of Plasma-Lyte 148 (Baxter Ptd Ltd, Australia) to a final volume of 1000 mL. The mixture was heparinized with 5000 units of unfractionated heparin and maintained under temperature conditions (23–28°C) using a benchtop incubator (Thermoline Pty Ltd, Australia) and agitated continuously. CRRT circuits were operated in CVVHDF mode with the following settings: blood flow rate of 20 mL/min, pre-blood pump fluid rate of 90 mL/h, bicarbonate-containing dialysis fluid flow rate of 100 mL/h and replacement fluid flow rate of 50 mL/h. The replacement fluid term refers to a technical fluid and flow rate that is required to prevent clotting in the bubble trap chamber in the CRRT filter circuit.^28^ The filtration was configured as pre-filter. Fluid removal rates were set at 200, 260 and 320 mL/h to simulate zero-flux (0 mL/kg/h or 0 mL/h), low-flux (20 mL/kg/h or 60 mL/h) and high-flux filtration (40 mL/kg/h or 120 mL/h), respectively, for a simulated 3 kg infant. The CRRT settings used for the blood flow rate and clearance in these simulations were those used in standard of care in the paediatric ICU at our institution and described in the literature.^29^ Each simulation was conducted over 30 minutes and repeated in triplicate. A schematic diagram of the closed-loop CRRT model and the sampling sites is shown in Figure S1 (available as Supplementary data at *JAC* Online).

#### Phase 2: adsorption study

For adsorption assessment, the method described by Sime and colleagues was adopted for the paediatric CRRT circuit.^30^ The mode of slow continuous ultrafiltration (SCUF) was selected, for the adsorption assessments to minimize diffusive clearance and isolate the antimicrobial loss attributable to circuit membrane adsorption. SCUF mode was used with an initial blood flow rate of 50 mL/min, which was subsequently reduced to 20 mL/min to maintain circuit flow stability. An ultrafiltration rate of 50 mL/h was applied, and no dialysis or replacement fluids were administered (i.e. flow rates were set to 0 mL/h). The effluent line was connected to a port in the ‘pseudo patient’ providing a recirculating closed-circuit system. Sodium chloride 0.9% was separately pumped into the effluent bag to prevent patient blood loss/gain alarms. Samples were collected over a 4-hour period and the antimicrobial recovery (%) was compared to the baseline (time zero). Antimicrobial recovery was used to estimate the potential for antimicrobial-circuit adsorption. All simulations were performed in triplicate.

### Control setup

Controls were prepared in triplicate using PVC jars containing 50 mL of human whole blood, 2500 units of unfractionated heparin (Pfizer Pty Ltd, Australia) and a single bolus of the study antimicrobials to achieve a final volume of ∼55 mL. The jars were maintained under the same temperature conditions (23–28°C) as the paediatric CRRT circuit using a benchtop incubator (Thermoline Pty Ltd, Australia) and were agitated continuously to ensure uniform antimicrobial distribution.

### Study antimicrobials preparation and administration

Once the CRRT circuit was stable, the study antimicrobials (ampicillin 500 mg, cefotaxime 500 mg, flucloxacillin 500 mg, fluconazole 100 mg, gentamicin 70 mg, meropenem 400 mg, micafungin 50 mg, piperacillin 1000 mg, tazobactam 125 mg, vancomycin 150 mg and voriconazole 90 mg) were added as a combined single bolus dose in various combinations (e.g. ampicillin, cefotaxime and meropenem) into the blood–crystalloid mixture. The PVC jars were incubated at 37^ο^C and agitated continuously to ensure uniform distribution of antimicrobials. Visual assessments indicated no observable interactions among the antimicrobials. The control preparation has been described in a previous *ex vivo* study.^31^ The physiochemical properties of the study antimicrobials are summarized in Table 1.^12–19^

### Sample collection

For assessment of clearance, pre- and post-filter and effluent samples (3 mL) were collected at baseline (0 minute) and at 30 minutes following each ultrafiltration rate change after the antimicrobial administration to determine the sieving coefficient and filter clearance of each antimicrobial in the CRRT simulation. Similarly, for adsorption assessment, samples (3 mL) were collected at baseline (0 minutes), and at ∼10, 30, 70, 120, 180 and 240 minutes after antimicrobial administration to determine antimicrobial recovery in the CRRT simulation. Corresponding control samples (3 mL) were collected at the same time points. Samples were immediately centrifuged at 3000**g** for 5 minutes. Plasma was separated and stored in cryovials at −80°C until analysis.

### Measurement of antimicrobials in plasma samples

Total plasma and effluent antimicrobial concentrations were measured using a validated high-performance liquid chromatography-tandem mass spectrometry (HPLC-MS/MS) method on a Nexera UHPLC system coupled to an 8030+ triple quadrupole mass spectrometer (Shimadzu, Kyoto, Japan). Calibration curves and quality control samples were included in each assay run, and all met predefined acceptance criteria. The precision for the plasma assays ranged from 3.7% to 9.1% with an accuracy ranging from 2.3% to 10.4%. For the effluent assays, the precision ranged from 3.3% to 6.3% and accuracy ranged from 1.5% to 15.6%.

### Calculations for the sieving coefficient, filter clearance and antimicrobial recovery

The sieving coefficient (Sc) for an antimicrobial was calculated using equation (1):


(1) $$Sc=\frac{\mathrm{Concentration}\;\mathrm{in}\;\mathrm{the}\;\mathrm{effluent}\;}{\mathrm{Concentration}\;\mathrm{in}\;\mathrm{the}\;\mathrm{plasma}\;}$$

The filter clearance for an antimicrobial was calculated using equation (2):


(2) $$\mathrm{Filter}\;\mathrm{clearance}\;(\mathrm{mL}/\min)=C_{\mathrm{effluent}}/C_{\mathrm{plasma}}\times Q_{\mathrm{effluent}}$$

where *Q*_effluent_ = dialysate rate + net ultrafiltrate rate.

The antimicrobial recovery demonstrated the antimicrobial-circuit adsorption this was calculated using equation (3):


(3) $$\text{Antimicrobial recovery}(\text{\%})=\left(\frac{\left(C_{t}\right)}{C_{i}}\right)\times 100))$$

where *C_t_* = plasma concentration at the time collected *t* and *C*_i_ = plasma concentration at the baseline time.

### Statistical analysis

One-way ANOVA was used to compare the means of antimicrobial sieving coefficients and filter clearances (mL/min) across three ultrafiltration rates (zero, low and high flux). Where significant differences were identified, Dunnett’s *post hoc* test was used to compare the ultrafiltration rates against zero flux. Linear regression analysis was used to compare the mean antimicrobial recovery (%) over time (minutes) between CRRT and control groups. Antimicrobial recovery (%) was modelled as a function of time, and group differences (CRRT versus control) were assessed by comparing the slopes of the regression lines. Antimicrobial recovery at 240 minutes were assessed using unpaired *t*-test. In addition, linear regression was used to evaluate associations between physicochemical characteristics (log *P*, molecular charge and protein binding) and the percentage of antimicrobial recovery at 240 minutes. All of the statistical analyses were performed using GraphPad Prism version 10 (GraphPad Software, Inc, La Jolla, CA, USA), with two-sided *P* value of <0.05 considered statistically significant.

## Results

The paediatric CRRT model and the control jars were maintained under comparable temperature conditions (mean temperature of 25.5 ± 4.8°C) for the blood–crystalloid mixture and the haematocrit was set at 0.3. A total of 109 samples per antimicrobial were analysed. Overall, there was no statistical difference identified in the simulations except for two simulations assessing clearance for ampicillin, cefotaxime and meropenem where we were unable to source ST 60 haemofilter, a ST 100 haemofilter was used instead with a membrane surface area of 1 m^2^.

The (mean ± SD) sieving coefficients and filter clearances for each antimicrobial across ultrafiltration rates (zero, low and high flux) are shown in Tables 2 and 3. The sieving coefficients for the antimicrobials did not vary significantly between zero flux, low flux and high flux (see Table 2). The filter clearance values were significantly higher in high flux compared with zero flux for fluconazole (*P* = 0.012), piperacillin (*P* = 0.01), tazobactam *P* = 0.003), vancomycin (*P* = 0.016) and voriconazole (*P* = 0.027) (see Table 3).

**Table 2.** Sieving coefficients for the antimicrobials at the ultrafiltrate rates

Antimicrobial	Sieving coefficient^a^, (*n* = 3)					
	No flux (0 mL/kg/h)		Low flux (20 mL/kg/h)		High flux (40 mL/kg/h)	
	Pre-filter	Post-filter	Pre-filter	Post-filter	Pre-filter	Post-filter
Ampicillin	1.06 ± 0.17	1.10 ± 0.05	1.05 ± 0.07	1.12 ± 0.08	0.94 ± 0.19	1.25 ± 0.38
Cefotaxime	0.89 ± 0.04	1.04 ± 0.13	0.99 ± 0.15	1.09 ± 0.13	1.07 ± 0.17	1.13 ± 0.23
Flucloxacillin	0.90 ± 0.22	0.96 ± 0.07	0.81 ± 0.14	0.80 ± 0.08	0.83 ± 0.09	0.63 ± 0.09
Fluconazole	0.92 ± 0.08	1.05 ± 0.04	0.96 ± 0.04	1.01 ± 0.07	0.98 ± 0.03	0.97 ± 0.08
Gentamicin	0.04 ± 0.01	BLQ	0.30 ± 0.14	BLQ	0.40 ± 0.12	BLQ
Meropenem	1.27 ± 0.11	1.31 ± 0.16	1.46 ± 0.24	1.32 ± 0.25	1.09 ± 0.05	1.51 ± 0.47
Micafungin	0.38 ± 0.04	0.30 ± 0.03	0.35 ± 0.09	0.23 ± 0.06	0.28 ± 0.03	0.15 ± 0.05
Piperacillin	0.91 ± 0.01	1.07 ± 0.06	1.03 ± 0.06	1.06 ± 0.08	1.14 ± 0.11	1.01 ± 0.04
Tazobactam	1.01 ± 0.08	1.1 ± 0.07	0.96 ± 0.06	1.09 ± 0.07	1.18 ± 0.11	1.19 ± 0.1
Vancomycin	0.88 ± 0.01	0.85 ± 0.02	0.90 ± 0.01	0.95 ± 0.07	0.86 ± 0.04	0.90 ± 0.07
Voriconazole	0.87 ± 0.02	0.81 ± 0.09	0.90 ± 0.09	0.91 ± 0.06	0.98 ± 0.06	0.87 ± 0.04

^a^Data expressed as mean ± SD.

BLQ, below the limit of quantification.

**Table 3.** Statistical analysis for the filter clearances for the antimicrobials at the ultrafiltrate rates, and percentage recovery (CRRT and control) at 240 minutes compared with baseline

Antimicrobial	Filter clearance^a^, mL/min (*n* = 3)			Group differences.^b^ (*P* value)	Recovery^a^ (%)	Control^a^ (%)	*P* value for slope comparison
	No flux	Low flux	High flux				
Ampicillin	5.4 ± 0.2	6.7 ± 0.5	8.7 ± 2.7	0.10	**49** **±** **2**^b^	**90** **±** **11**^b^	**0**.**048**
Cefotaxime	5.2 ± 0.6	6.5 ± 0.8	7.9 ± 1.6	0.07	54 ± 2	**85** **±** **3**^b^	0.470
Flucloxacillin	4.8 ± 0.4	4.8 ± 0.5	4.4 ± 0.7	0.53	43 ± 1	**88** **±** **6**^b^	0.374
Fluconazole	5.3 ± 0.1	6.0 ± 0.5	**6.8** **±** **0.5**^b^	**0**.**01**	**76** **±** **4**^b^	98 ± 5	**0**.**033**
Gentamicin	7.0 ± 0.8	25.2 ± 12.6	34.8 ± 21.6	0.13	0 ± 0	**91** **±** **3**^b^	**<0.001**
Meropenem	6.6 ± 0.7	7.9 ± 1.5	10.6 ± 3.3	0.15	**51** **±** **4**^b^	**79** **±** **5**^b^	0.092
Micafungin	1.5 ± 0.2	1.4 ± 0.4	1.1 ± 0.3	0.27	90 ± 4	102 ± 2	0.386
Piperacillin	5.2 ± 0.2	6.2 ± 0.8	**7.0** **±** **0.2**^b^	**0**.**01**	**54** **±** **1**^b^	**96** **±** **2**^b^	**0**.**033**
Tazobactam	5.7 ± 0.1	6.5 ± 0.3	**7.4** **±** **0.5**^b^	**< 0.01**	61 ± 2	**96** **±** **4**^b^	0.408
Vancomycin	4.8 ± 0.4	5.4 ± 0.3	**6.0** **±** **0.3**^b^	**0**.**01**	**31** **±** **2**^b^	98 ± 4	**<0.001**
Voriconazole	4.4 ± 0.3	5.4 ± 0.4	**5.6** **±** **0.5**^b^	**0**.**01**	**47** **±** **2**^b^	99 ± 3	**<0.001**

^a^Data expressed as mean ± SD.

^b^Bold indicates significant *P*-value of  < 0.05.

**Figure 1.**
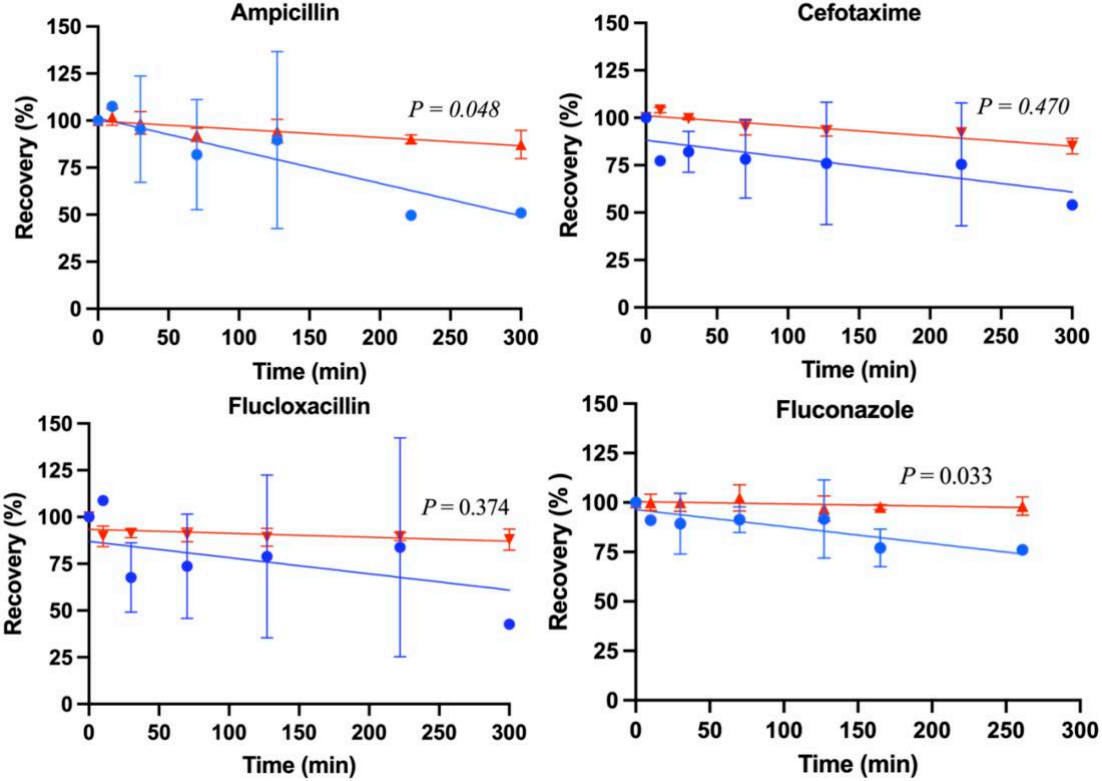
Percentage of recovery in the paediatric CRRT model over time (min), for ampicillin, cefotaxime, flucloxacillin and fluconazole, with the mean SD for RRT (blue) and control (red) and a line of best fit. *P* value for the slope comparison with *P* < 0.05 being significant.

**Figure 2.**
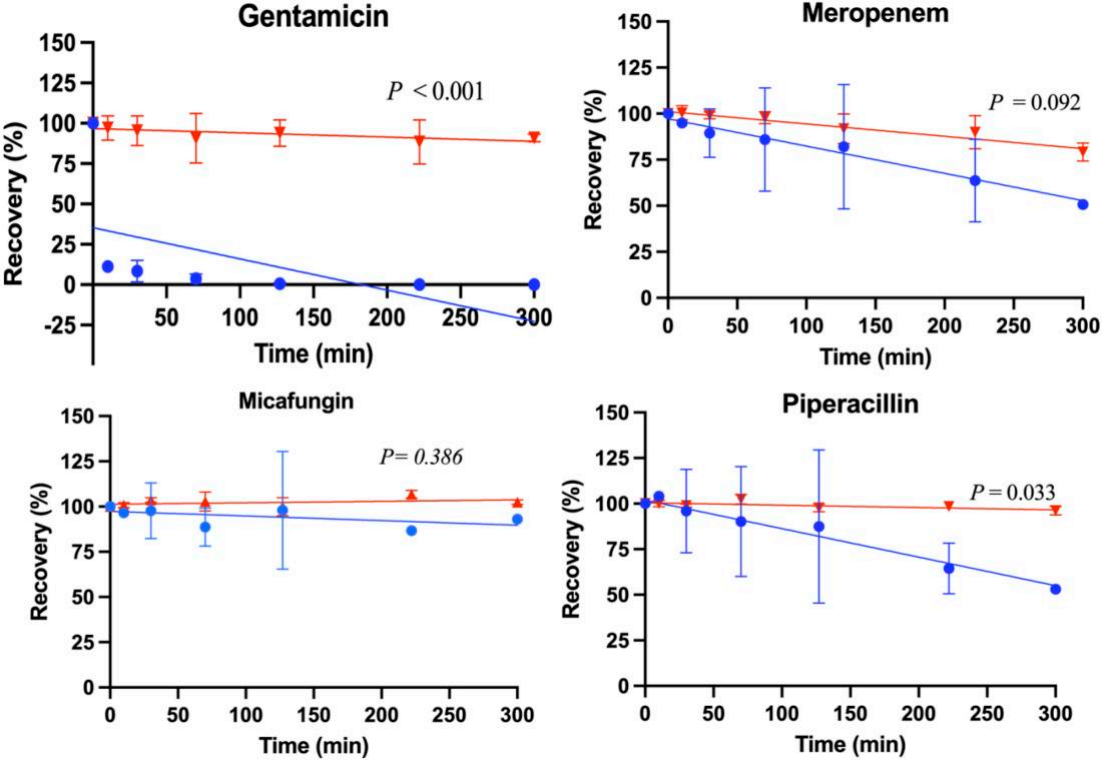
Percentage of recovery in the paediatric CRRT model over time (min), for gentamicin, meropenem, micafungin and piperacillin, with the mean SD for RRT (blue) and control (red) and a line of best fit. *P* value for the slope comparison with *P* < 0.05 being significant.

**Figure 3.**
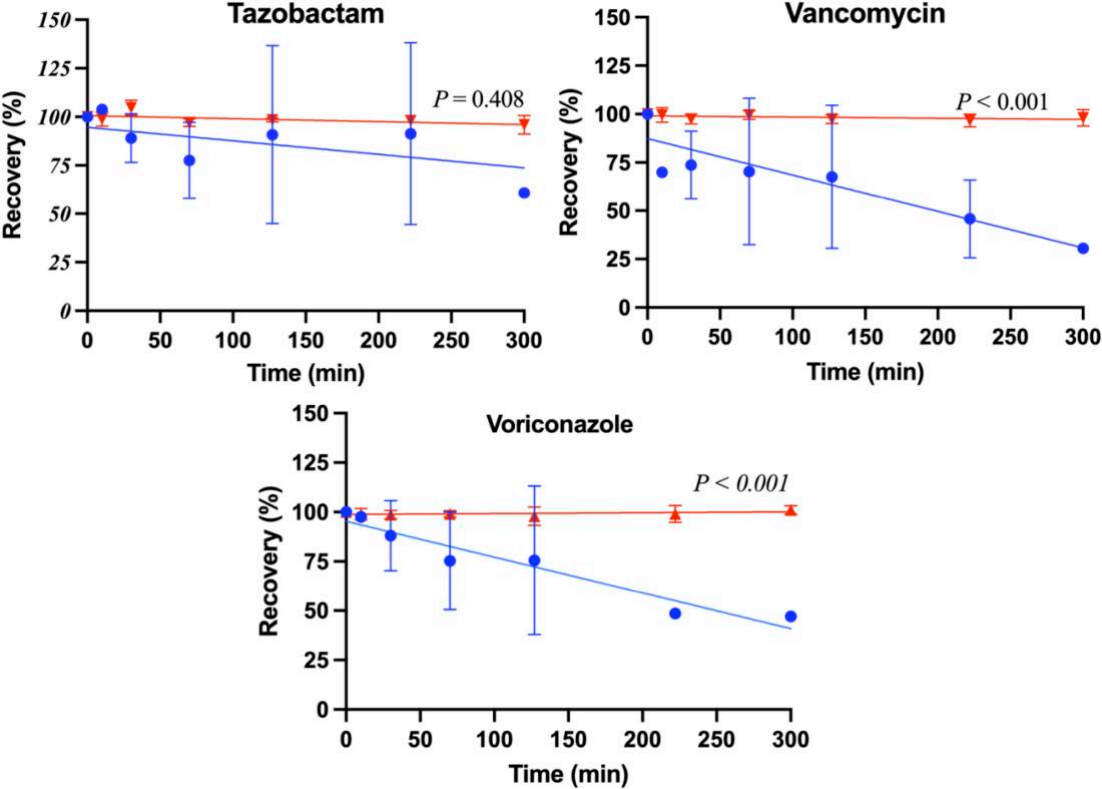
Percentage of recovery in the paediatric CRRT model over time (min), for tazobactam, vancomycin and voriconazole, with the mean SD for RRT (blue) and control (red) and a line of best fit. *P*-value for the slope comparison with *P* < 0.05 being significant.

The percentage recovery of each antimicrobial (mean ± SD) was modelled as a function of time (minutes) for both CRRT circuits and control groups, as shown in Figures 1–3. The mean (± SD) antimicrobial recovery at 240 minutes relative to baseline in CRRT circuits and controls is summarized in Table 3. In the CRRT circuits, mean recoveries were significantly lower than baseline for ampicillin (*P* = 0.045), fluconazole (*P* = 0.03), meropenem (*P* = 0.02), piperacillin (*P* = 0.02), vancomycin (*P* = 0.01) and voriconazole (*P* < 0.01). In the control groups, significant reductions from baseline were observed for ampicillin (*P* < 0.001), cefotaxime (*P* < 0.001), flucloxacillin (*P* = 0.024), gentamicin (*P* = 0.02), meropenem (*P* < 0.001), piperacillin (*P* = 0.003) and tazobactam (*P* = 0.016). When comparing CRRT circuits with controls, mean recoveries were significantly different for ampicillin (*P* = 0.048), fluconazole (*P* = 0.033), gentamicin (*P* < 0.001), piperacillin (*P* = 0.033), vancomycin (*P* = 0.001) and voriconazole (*P* < 0.001).

**Figure 4.**
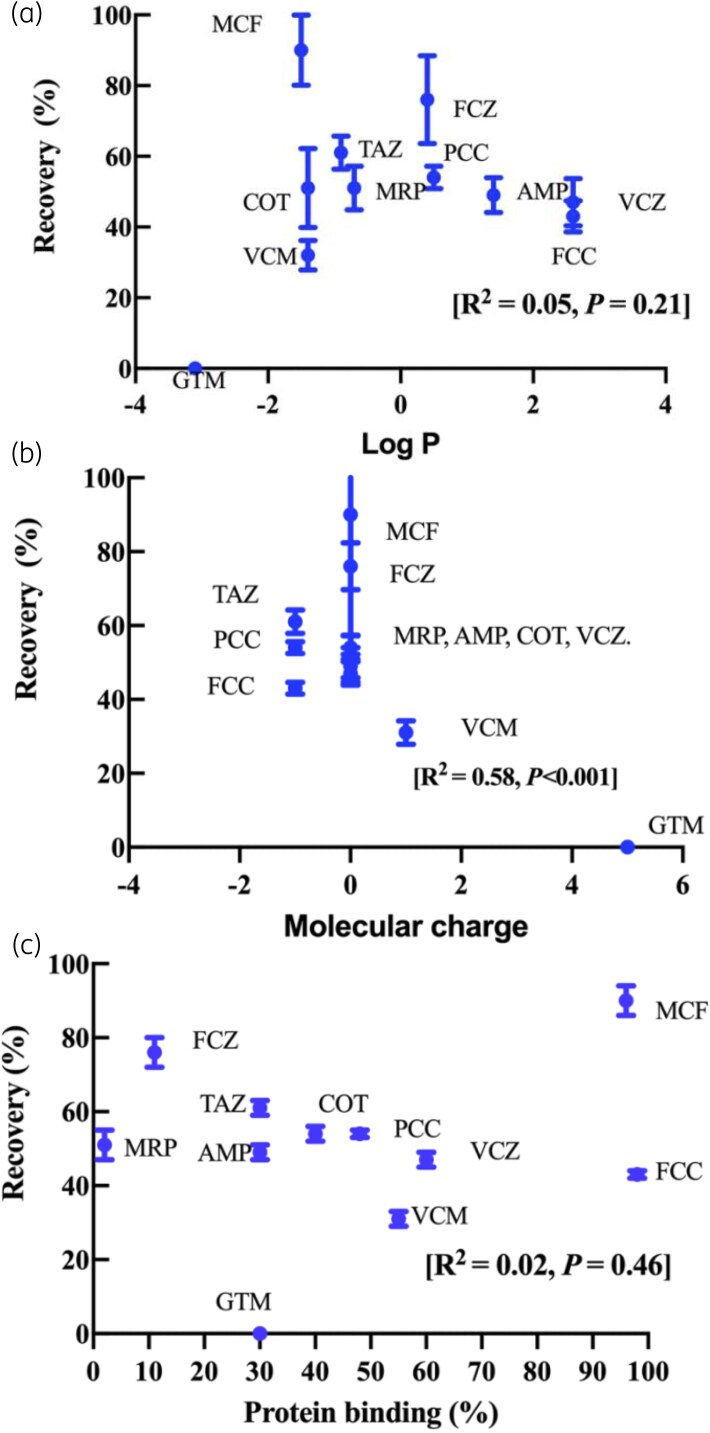
Recovery of antimicrobials (%) in the CRRT at 240 minutes compared with baseline. (a) Lipophilicity expressed as log partition coefficient (log *P*) values, (b) molecular charge and (c) protein binding (%). For each drug, the mean recovery is indicated by a circle and the upper and lower 95% confidence intervals are indicated by crossbars. AMP, ampicillin; COT, cefotaxime; FCC, flucloxacillin; FCZ; fluconazole, GTM, gentamicin; MRP, meropenem; MCF; micafungin, PCC, piperacillin; TAZ, tazobactam; VCM, vancomycin, VCZ; voriconazole.

The relationships between lipophilicity (log *P*), molecular charge and protein binding (%) with antimicrobial recovery (%) in the CRRT circuit at 240 minutes are presented in Figure 4.

A significant relationship was observed between molecular charge (*R*^2^ = 0.57, *P* < 0.0001) and antimicrobial recovery. No significant relationships were reported between antimicrobial recovery and log *P* (*R*^2^ = 0.05, *P* = 0.211) or protein binding (*R*^2^ = 0.03, *P* = 0.375).

## Discussion

This study is the largest *ex vivo* investigation to date evaluating the impact of a paediatric CRRT configuration on 11 commonly used antimicrobials. Reduced antimicrobial concentrations were demonstrated in >70% of the study antimicrobials, driven by either an increased filter clearance or antimicrobial-circuit adsorption (see Table 3 and Figures 1–3). A significant relationship was identified between the molecular charge of the antimicrobial and the antimicrobial recovery (see Figure 4) indicating that the physicochemical properties could be used to predict the extent of antimicrobial loss in a paediatric CRRT treatment. These findings are of clinical significance for critically ill children receiving CRRT where TDM is not available for all antimicrobials, and the current dosing strategies may lead to under-dosing of antimicrobials. Our findings were also compared with published *ex vivo* studies, and although differences were reported, these were predominantly due to the use of adult CRRT models with different circuit compositions and filter characteristics (see Table S1^20–25,29,30,32–36^)(availabe as Supplementary data at JAC Online).

The sieving coefficient describes the ability of a drug to pass through the CRRT filter membrane and can be estimated from the drug’s plasma protein binding. However, in critically ill patients, protein binding is highly variable due to altered physiology and hypoalbuminemia,^37^ which may be further altered in a critically ill child from organ maturation.^38^ Antimicrobials with a high molecular weight (>500 Daltons) and high protein binding (>70%) are generally not expected to be filtered by CRRT.^8–11^ A sieving coefficient of approaching or >1 indicates extensive passage of the drug across the CRRT membrane.^30,39,40^ In this *ex vivo* study, sieving coefficients of >1 were observed for ampicillin (0.94–1.25), cefotaxime (0.89–1.13), meropenem (1.09–1.51), piperacillin (0.91–1.14) and tazobactam (0.96 −1.19), suggesting substantial filtration of these antimicrobials during CRRT, which can potentially lead to sub-therapeutic antimicrobial concentrations in the critically ill child. Similar sieving coefficients have been reported in previous adult *ex vivo* CRRT studies for cefotaxime (0.9–1),^34^ meropenem (1.06–1.53),^20,24,30^ piperacillin (0.7–1.02)^20,41^ and tazobactam (0.78–1.04)^41^. In our *ex vivo* study, ultrafiltration rates did not significantly influence the sieving coefficients for these antimicrobials. An adult study in critically ill hypoalbuminemia patients receiving CRRT identified a relationship between the sieving coefficient and antimicrobial fraction unbound to albumin,^42^ suggesting variability is likely in the critically ill patient. The sieving coefficients for the study antimicrobials were reported to be in the range for the antimicrobials.^34,42,43^ Our findings indicate the need for paediatric specific studies as extrapolation from adult models or studies may not reflect paediatric scenarios.

High flux (CRRT of 40 mL/kg/h) is commonly used in critically ill paediatric patients. In our study, this setting led to significantly increased filter clearance for fluconazole, gentamicin, piperacillin, tazobactam, vancomycin and voriconazole (*P* < 0.05) (see Table 3). A clinical study in critically ill adults receiving gentamicin (7 mg/kg every 24 hours) during high flux (CRRT of 40 mL/kg/h) demonstrated target attainment (MIC ≥1 mg/L),^44^ although current recommendations advocate dose reduction to 2−5 mg/kg every 24 hours.^45^ Similarly, a study investigating vancomycin in critically ill adults receiving high flux (CRRT >50 mL/kg/h) reported sub-therapeutic concentrations.^46^ In our study, filter clearances for gentamicin (35 mL/min) and vancomycin (6 mL/min) were increased under high flux (CRRT of 40 mL/kg/h). Similarly, the mean filter clearances for meropenem (8.3 mL/min) and piperacillin (6.1 mL/min) were comparable to those reported in an *ex vivo* study for meropenem of 7.7 mL/min and piperacillin of 5 mL/min.^29^ By contrast, another *ex vivo* study using an adult CRRT model with a higher blood flow rate (200 mL/min) and a larger polysulfone haemofilter (1.2 m^2^) reported significantly greater filter clearances for meropenem (19.5 mL/min) and piperacillin (16 mL/min).^20^ These findings suggest that the filter composition, blood flow rate and CRRT modality substantially influence the antimicrobial clearance. In our simulations, high-flux CRRT (40 mL/kg/h) demonstrated increased clearance in several of the antimicrobials, which may contribute to sub-therapeutic concentrations in critically ill children receiving CRRT.

Factors that may affect the drug-circuit adsorption are the drug’s physiochemical properties (log *P*, molecular charge and protein binding) and the composition of the circuit.^29,35^ In our study, overall, there was 70% reduced concentrations, with 60% of the antimicrobials reporting significantly reduced recovery from drug-circuit adsorption at 240 minutes. Linear regression was used as a descriptive approach to compare overall recovery trends between CRRT circuits and controls rather than to model this adsorption process. A significant relationship between the molecular charge and antimicrobial recovery supports previous findings that positively charged drugs, such as gentamicin and vancomycin, may bind to negatively charged AN69ST filter membranes^35^ (see Figures 2 and 3). This is consistent with previous *ex vivo* studies demonstrating a recovery of 10% for gentamicin and 45% for vancomycin at similar observation time periods.^22^ In addition, voriconazole demonstrated substantial loss in our study, which aligns with a clinical study in adults receiving CRRT that attributed the reduced voriconazole concentrations to both drug-circuit adsorption and high-flux clearance.^47^ The extent of drug-circuit adsorption appears to be filter-specific, with prior studies demonstrating that larger surface areas (>1.5 m^2^)^23–25,33,34^ and different filter compositions (polyacrylonitrile haemofilter, asymmetric triacetate haemofilter or cytokine adsorbent systems)^21,24,25^ were associated with decreased antimicrobial recoveries (see Table 3^20–25,29,30,32–36^). Studies performed in paediatric *ex vivo* CRRT circuits that describe antimicrobial’s adsorption are limited, however, studies such as our *ex vivo* study may inform the extracorporeal clearance for the individual antimicrobial during CRRT. Clinical studies are required to determine the clinical significance of these findings in critically ill children receiving CRRT.

Previous studies have reported spontaneous antimicrobial degradation in crystalloid solutions for ampicillin, meropenem and piperacillin,^31,36,48^ leading to potentially higher filter clearances or increased circuit adsorption. For our *ex vivo* study, the blood volume used for the blood–crystalloid mixture reflected the blood volume of an infant and this blood to crystalloid ratio may reflect differences in antimicrobial-circuit adsorption when results are compared to adult *ex vivo* CRRT studies.^20,30,32,33,36,49^ Similarly, the control was primarily blood and the antimicrobial recovery over time may differ in the CRRT simulations due to the blood–crystalloid ratio. A blood volume of 300 mL was selected to approximate the circulating blood volume of a 3 kg infant. However, this blood to crystalloid ratio may have reduced plasma protein concentrations and increased the unbound fraction of highly protein bound antimicrobials such as flucloxacillin and micafungin. The sieving coefficients observed in this *ex vivo* study for flucloxacillin (0.63–0.96) and micafungin (0.15–0.38) were higher than those previously reported for flucloxacillin (mean 0.3)^43,50^ and micafungin (<0.01).^36^ Therefore, these findings should be cautiously interpreted for highly protein bound antimicrobials. The antimicrobial recoveries in our study were compared to adult *ex vivo* studies, identifying a lower recovery for fluconazole (76% versus 100%),^33^ gentamicin (0% versus 5%),^22,25^ meropenem (51% versus 85%),^20,29,30,36^ piperacillin (54% versus 74%),^20,32^ vancomycin (31% versus 45%)^25^ and voriconazole (47% versus 73%).^29^ These reported differences in the antimicrobial recoveries may be a result of an increased filter surface area (1.2–1.8 m^2^) or different filter composition (acrylonitrile and sodium methallyl sulfonate, polyacrylonitrile, polycarbonate/polyurethane and polyethersulfone)^20,25,32^ or a shorter observation time (0.33 to 3 hours). Previous *ex vivo* studies have identified that the filter composition will result in differ for fluconazole (95% versus 100%),^33^ gentamicin (<10% versus 100%^22^ and 13% versus 100%),^25^ meropenem (88% versus 97%)^36^ and micafungin (79% versus 99%).^36^ The filter composition has a significant role in antimicrobial recovery. In addition, the blood flow rates used in critically ill children receiving CRRT range from 15 to 200 mL/min while the blood flow rates for adults range from 100 to 450 mL/min and may result in differ extracorporeal clearance for drugs. Different filter compositions, surface area, blood blow rates and observation times may result in different filter clearance and drug-circuit adsorption for antimicrobials. However, for many antimicrobials evaluated in this study, including ampicillin, flucloxacillin, fluconazole and voriconazole, there are no published paediatric *ex vivo* studies using comparable filter compositions. This highlights a major evidence gap in antimicrobial dosing guidance for children receiving CRRT.

This study has several limitations. The blood–crystalloid mixture does not replicate the physiology of a critically ill child, and the circuit volumes used in the *ex vivo* paediatric CRRT model do not reflect clinical scenarios. The absence of anticoagulant metabolism in the circuit prevents clot regulation, leading to ongoing haemolysis that can alter antimicrobial plasma concentrations.^51^ The haematocrit for this *ex vivo* study was set at 0.3. In critically ill patients the haematocrit is highly variable resulting in variable antimicrobial plasma concentrations.^52^ The blood–crystalloid composition ratio used in our *ex vivo* study may have reduced plasma protein binding potentially increasing the unbound fraction of the highly protein bound antimicrobials and resulting in higher measured sieving coefficients for the antimicrobials. An adult *ex vivo* study demonstrated higher sieving coefficients for micafungin and piperacillin and tazobactam when a crystalloid solution was used instead of human plasma in as *ex vivo* RRT circuit.^36^ In addition, the controls did not contain the same blood–crystalloid ratio as the CRRT circuit, and this may limit comparative antimicrobial recovery. For two extracorporeal clearance simulations involving ampicillin, cefotaxime and meropenem, a ST 100 haemofilter with 1 m2 membrane surface area was used, instead of the ST60 haemofilter. Therefore, clearance estimates for these antimicrobials may not be directly comparable. This study did not describe the impact of repeated doses of antimicrobials on the filter membrane, where a study investigating repeated gentamicin doses identified less reduced concentrations for gentamicin.^23^ Importantly, the manufacturers of the CRRT filters recommend the filters to be replaced every 72 hours. In our study, the antimicrobials were added as a single bolus and should not be extrapolated for extended or continuous infusions used in the clinical setting, although the extracorporeal clearance and adsorption is relevant it may result in less pronounced concentration losses. This study assessed the impact of the filters commonly used for critically ill children, and alternative filters may demonstrate different antimicrobial recoveries (see Table S1^20–25,29,30,32–36^)(available as Supplementary data at JAC Online). Different CRRT modalities may influence the antimicrobial-circuit adsorption. In this study adsorption was assessed using SCUF, whereas CVVHDF is commonly used in the clinical practice that may limit the interpretation of the findings. In addition, the composition of the reservoir (blood–crystalloid mixture) may also alter the antimicrobial clearance. A buffered solution containing human serum albumin demonstrated higher clearances for vancomycin,^35^ which may be associated with vancomycin’s protein binding. Several antimicrobial recovery findings reported marginal statistical significance with *P* values close to 0.05. Given the exploratory nature of this study and the limited number of simulations these findings should be interpreted cautiously. Further simulations would improve the precision of the estimates and help confirm the reproducibility of these findings. A recent clinical study identified that using *ex vivo* models assisted in the development of optimal dosing regimens for antimicrobials in adults receiving CRRT.^53^ Future developments need to determine whether the current antimicrobial regimens provide therapeutic concentrations in critically ill children receiving CRRT.

### Conclusions

In this *ex vivo* study, reduced antimicrobial concentrations were identified in >70% of the study antimicrobials, attributable to either an increased clearance from high-flux (CRRT dose >40 mL/kg/h) or antimicrobial-circuit adsorption. Among the physicochemical properties assessed, the molecular charge of an antimicrobial was found to be a key predictor of antimicrobial recovery, whereas a relationship was not found for log*P* and protein binding. These findings suggest that CRRT in critically ill children may contribute to sub-therapeutic antimicrobial exposures, potentially increasing the risk of treatment failure and antimicrobial resistance. The findings lend further importance to the need for affordable rapid TDM solutions for main antimicrobials to ensure adequate dosing. Further clinical studies are required to determine the significance of these findings and to assess whether current antimicrobial dosing regimens achieve therapeutic concentrations in critically ill children receiving CRRT.

## Supplementary Material

dkag189_Supplementary_Data

## Supplementary data

Figure S1 and Table S1 are available as Supplementary data at *JAC* Online.
