# Supplementary material for: The impact of an ex vivo paediatric renal replacement therapy circuit on antimicrobial concentrations
Source: J Antimicrob Chemother. 2026 Jun 3;81(7):dkag189. doi: 10.1093/jac/dkag189 (PMC13232042; doi:10.1093/jac/dkag189)
Supplement: dkag189_Supplementary_Data [file dkag189_supplementary_data.pdf]

Supplementary Table 1: Recovery of antimicrobials in *ex-vivo* RRT studies over time

Supplementary Figure 1: Schematic of the *ex-vivo* paediatric CRRT model with highlighted samples points (pre- and post- filter & effluent highlighted in green).

Study	Duration of laboratory (h)	Type of RRT filter	Surface Area (m^2)	Percent remaining (%)									
				Ampicillin	Cefotaxime	Flucloxacillin	Fluconazole	Gentamicin	Meropenem	Micafungin	Piperacillin	Vancomycin	Voriconazole
**Study**	**4**	**AN69ST hollow fibre**	**0.6**	**49**	**54**	**43**	**76**	**0**	**51**	**90**	**54**	**31**	**47**
Lam[a] 2010 [1]	0.33	PAN	0.6	NR	NR	NR	NR	<10	NR	NR	NR	NR	NR
Lam[a] 2010 [1]	0.33	Polyamide	0.6	NR	NR	NR	NR	100	NR	NR	NR	NR	NR
Jamal[b] 2015 [2]	4	Aquamax (polysulfone)	1.2	NR	NR	NR	NR	NR	94	NR	91	NR	NR
Sime[b] 2018 [3]	2.5	AN69ST100	1	NR	NR	NR	NR	NR	85	NR	NR	NR	NR
Purohit[a], 2019 [4]	3	HF 1000	1.1	NR	NR	NR	NR	NR	85	NR	74	NR	73
Economou[b], 2020 [5]	4	PES	1.2	NR	NR	NR	NR	NR	NR	NR	80	NR	NR
Baud[b], 2021 [6]	6	ST150 PAN	1.5	NR	NR	NR	NR	5	NR	NR	NR	NR	NR
Baud[b], 2021 [7]	6	AN69ST hollow fibre	1.5	NR	NR	NR	100	NR	NR	NR	NR	NR	NR
Baud[b], 2021[7]	6	AV 1000 PS	1.8	NR	NR	NR	95	NR	NR	NR	NR	NR	NR
Onichimowski[b] 2021 [8]	3	PAN PEI	1.5	NR	NR	NR	NR	13	NR	NR	NR	55	NR
Onichimowski[b] 2021[8]	3	AV 1000 PS	1.8	NR	NR	NR	NR	100[b]	NR	NR	NR	50	NR
Kortge[b], 2021[9]	6	ATA	1.5	NR	NR	NR	NR	NR	2	NR	NR	6	NR

Le Ven[b], 2023 [10]	5.5	ST150 PAN	1.5	NR	72	NR	NR	NR	NR	NR	NR	NR	NR
Le Ven[b] 2023 [10]	6	AV 1000 PS	1.8	NR	71	NR	NR	NR	NR	NR	NR	NR	NR
Inano[a] 2024 [11]	0.33	AN69ST hollow fibre	0.8	NR	NR	NR	NR	NR	NR	NR	NR	45	NR
McGrath[b] 2025 [12]	2	HF1400	1.4	NR	NR	NR	NR	NR	88 to 96	79 to 87	NR	NR	NR
McGrath[b] 2025 [12]	2	AN69M100	0.9	NR	NR	NR	NR	NR	91 to 97	93 to 99	NR	NR	NR

Bold represents our study. AN69ST acrylonitrile & sodium methallyl sulfonate copolymer haemofilter membrane; surface treatment (ST) of polyethylene imine; [a] paediatric circuitry; PAN polyacrylonitrile haemofilter membrane; NR not reported; [b] adult circuitry; Cytosorb cytokine adsorbent system; HF 1000 polyarylethersulfone haemofilter membrane, PES polyethersulfone; AV arteriovenous; PS polysulfone haemofilter membrane; ST polycarbonate/polyurethane; PEI surface treatment of polyethyleneimine; ATA asymmetric triacetate haemofilter membrane.

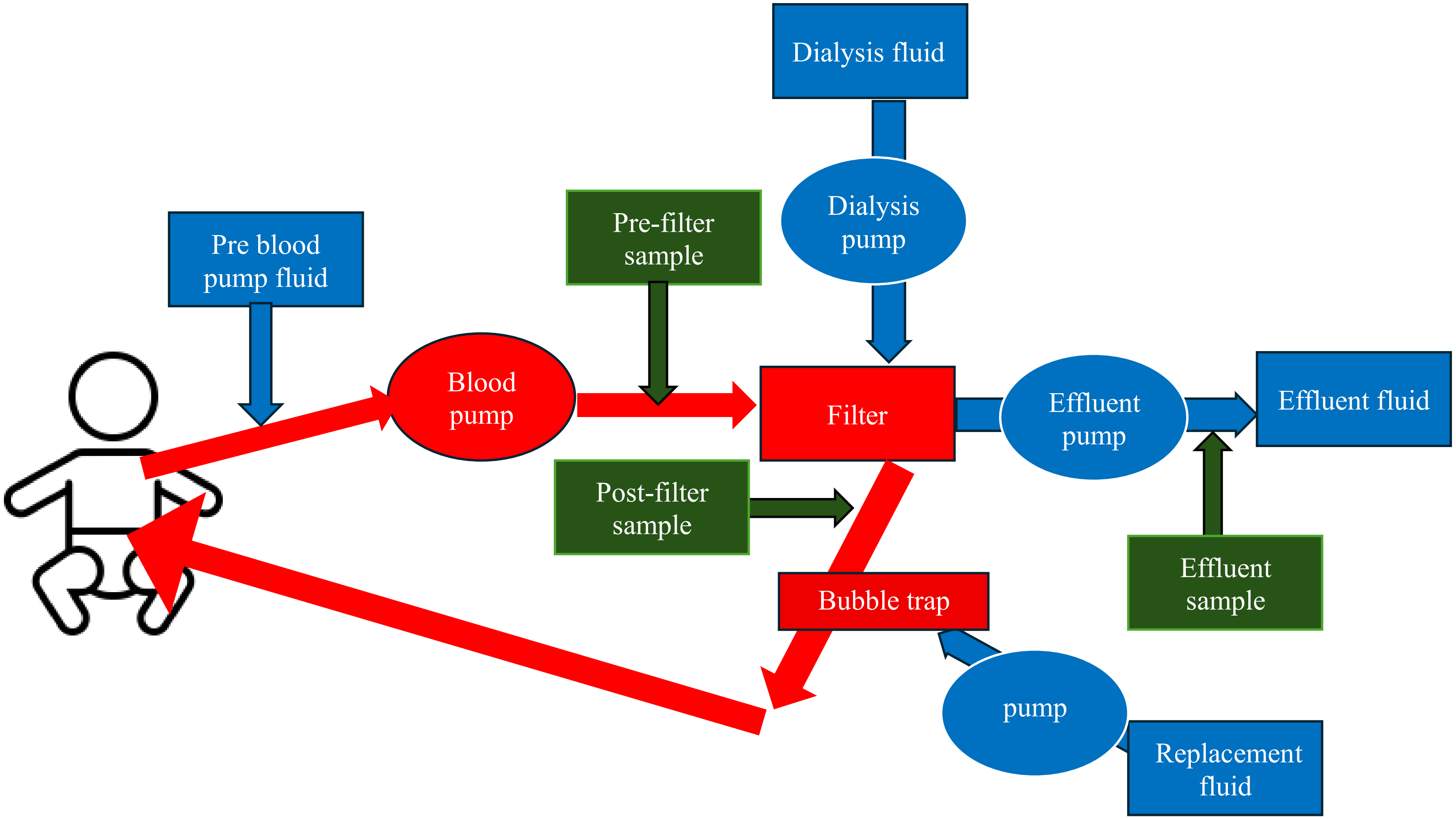
Dialysis fluid
Dialysis pump
Pre-filter sample
Pre blood pump fluid
Blood pump
Filter
Effluent pump
Effluent fluid
Post-filter sample
Bubble trap
Effluent sample
pump
Replacement fluid
